# Tetanus

**Published:** 1900-12-01

**Authors:** 


### TETANUS.

Mr. A. E. Barker [1] reports a case of tetanus in which subdural injection of anti-tetanic serum was employed and was followed by recovery. The symptoms came on ten days after the injury, and on the fourth day of the symptom he was admitted to hospital. The skull was trephined with a quarter-inch trephine, the needle of the syringe was passed into the subdural space, and 7½ cubic centimetres of anti-tetanic serum, prepared at the Jenner Institute, were injected. During the four following days 20 cubic centimetres of serum were injected into the flanks. Gradually increasing doses of chloral also were administered until the patient was taking 80 grains per diem. No improvement took place for a week, when the rigidity of the muscles of the neck and abdomen began to pass off. At the end of three weeks from his admission all the stiffness had gone. That in the legs lasted the longest. Dr. Sydney Long [2] also reports a case of acute (traumatic) tetanus treated with antitetanus serum. The treatment was not commenced until seven clear days after the establishment of the disease. The antitoxin was administered at first hypodermically and afterwards per rectum.

[1] Lancet, Nov. 17. [2] Brit. Med. Jour., Nov. 24.

